# *Saccharomyces boulardii* CNCM I-745 Stimulates Intracellular Antimicrobial Activity Against *Salmonella* Typhimurium in Murine Macrophages 

**DOI:** 10.3390/microorganisms14040787

**Published:** 2026-03-31

**Authors:** Rodolphe Pontier-Bres, Dorota Czerucka

**Affiliations:** Biomedical Department, Centre Scientifique de Monaco, 8 quai Antoine 1^er^, 98000 Monaco, Monaco; rpontier-bres@centrescientifique.mc

**Keywords:** *Salmonella* Typhimurium, probiotic, *Saccharomyces boulardii* CNCM I-745, antimicrobial activity, macrophage, iNOS

## Abstract

*Salmonella enterica* serovar Typhimurium (ST) is an intracellular pathogen that survives within macrophages and disseminates to systemic organs, thereby evading host immune defenses. Previous studies have shown that the probiotic yeast *Saccharomyces boulardii* CNCM I-745 improves survival in ST-infected mice, reduces bacterial translocation, and modulates cytokine expression, including the upregulation of interferon-γ and the downregulation of interleukin-10, both of which are involved in the regulation of inducible nitric oxide synthase (iNOS), a key mediator of macrophage antimicrobial activity. The present study was designed to investigate the transcriptional regulation of iNOS and associated antimicrobial responses in ST-infected RAW264.7 murine macrophages pretreated or cotreated with *S. boulardii*. Gene expression levels of iNOS and selected cytokines were analyzed in RT-qPCR assays. Bacterial adhesion was quantified by colony-forming unit (CFU) counting, and intracellular survival was assessed using a gentamicin protection assay. *S. boulardii* did not affect bacterial adhesion, but it significantly reduced intracellular ST survival, particularly under pretreatment conditions (*p* < 0.05). This effect was associated with increased iNOS gene expression. Interferon-γ expression was mainly induced by pretreatment, whereas tumor necrosis factor-α and interleukin-10 were modulated under cotreatment conditions. These findings indicate that *S. boulardii* modulates macrophage antimicrobial gene expression and suggest that probiotic pretreatment enhances innate immune responses against intracellular bacterial infections.

## 1. Introduction

Probiotics (defined by the United Nations Food and Agriculture Organization as “live microorganisms which, when administered in adequate amounts, confer a health benefit on the host”) have been extensively studied for their roles in maintaining intestinal homeostasis, modulating inflammation, and enhancing immune responses [1,2,3]. Although the most frequently consumed probiotics include lactic-acid-producing bacteria of human origin (such as *Lactobacillus* and *Bifidobacterium* species [4,5]), probiotic yeasts have also demonstrated significant clinical benefits. In particular, the CNCM I-745 strain of *Saccharomyces boulardii* (a yeast originally isolated from tropical fruit peel) has been widely used as a probiotic in human medicine [6]. Numerous clinical studies and systematic reviews support the use of *S. boulardii* not only in patients with chronic diseases (such as Crohn’s disease and irritable bowel syndrome) but also in those with acute conditions (including antibiotic-associated diarrhea, enteral-nutrition-related diarrhea, and traveler’s diarrhea) [6,7,8]. In the context of acute pathogen-induced diarrhea, *S. boulardii* exerts beneficial effects through multiple mechanisms, including binding bacterial pathogens to its cell wall, inhibiting bacterial motility, neutralizing toxins, and modulating pathogen-induced host signaling pathways [9].

Furthermore, *S. boulardii* has been shown to modulate innate and adaptive immune responses in mouse models of inflammation and infection [10,11]—contexts in which the yeast’s interaction with innate immune cells originating from mice and humans have been investigated [12,13,14]. As the first-line sentinel immune cells in the lamina propria, macrophages have a key role in clearing bacteria (including *Mycobacterium tuberculosis*, *Legionella pneumophila*, *Brucella* species, and *Salmonella enterica* subspecies *enterica* serovar Typhimurium) from infected tissues [15]. Macrophages can deploy several antimicrobial mechanisms, one of which is the production of potent, reactive nitrogen intermediates synthesized by inducible nitric oxide synthase [16,17]. Inducible nitric oxide synthase (iNOS) expression in macrophages is regulated tightly by the presence of microbial products and cytokines [18]. Via the NF-κB and STAT1 signaling pathways, pro-inflammatory mediators such as interferon gamma (IFN-γ), tumor necrosis factor alpha (TNF-α), and interleukin (IL)-1 beta (IL-1β) act synergistically to promote the transcription of the gene coding for iNOS [18,19,20]. In contrast, anti-inflammatory cytokines (such as IL-10) suppress iNOS expression and help to maintain immune homeostasis [21,22]. This balance in cytokine signaling ultimately determines the level of nitric oxide (NO) production and the effectiveness of microbial clearance mechanisms.

*Salmonella enterica* subspecies *enterica* serovar Typhimurium (ST) is a major foodborne pathogen in humans; it is responsible for diseases ranging from mild gastroenteritis to severe enterocolitis [23]. Although mice are naturally resistant to ST and develop a typhoid-like syndrome with low intestinal colonization, prior treatment with an antibiotic (e.g., streptomycin) depletes the resident intestinal microbiota, facilitates ST colonization and translocation, and thus creates a model mimicking the human infection [24]. ST uses a type III secretion system—a specialized molecular apparatus found in Gram-negative bacteria—to inject effector proteins into host cells [23,25]. The secretion system encoded by *Salmonella* pathogenicity island 1 (SPI-1) is essential for epithelial invasion and diarrhea induction, while that encoded by SPI-2 is crucial for systemic infection by allowing ST to survive and replicate within macrophage vacuoles and thus evade NO-mediated killing [25]. Hence, iNOS activity is essential for limiting ST proliferation in host organs, as demonstrated by the elevated mortality rate observed in iNOS knock-out mice challenged with ST [26,27].

Previous research in the mouse has shown that *S. boulardii* can attenuate the severity of ST infection via several mechanisms [28,29]. In a typhoid-like model, *S. boulardii* administration was associated with a lower mortality rate and prevented bacterial dissemination to the liver [28]. In the streptomycin-pretreated model, *S. boulardii* was associated with a lower degree of intestinal inflammation and less translocation of ST to the liver and spleen [13,29]. These effects were accompanied by an immunomodulatory shift, with the recruitment of tissue-resident macrophages, elevated systemic IFN-γ levels, and lower IL-10 levels in the gut [13,29]. This modulation of the cytokine profile might upregulate iNOS expression by macrophages. However, the direct impact of *S. boulardii* on macrophage antimicrobial activity in general and on iNOS activation in particular has not previously been characterized.

The RAW264.7 murine macrophage-like cell line is a well-established model of ST-macrophage interactions, especially in relation to hypoxia and iNOS responses [30,31]. The primary objective of the present study was to assess the effects of *S. boulardii* pretreatment and cotreatment protocols (see below) on the antimicrobial activity of RAW264.7 macrophage-like cells infected with ST *in vitro*.

## 2. Materials and Methods

### 2.1. Cell Culture and Microorganisms

The murine macrophage-like cell line RAW264.7 (ATCC) was cultured in Dulbecco’s Modified Eagle Medium (DMEM, Life Technologies, Courtaboeuf, France) supplemented with 10% heat-inactivated fetal bovine serum (FBS; HyClone, Cytiva, Saint-Germain-en-Laye, France), 2 mM L-glutamine, and antibiotics (100 U/mL penicillin and 100 µg/mL streptomycin; Gibco, Bourgoin-Jallieu, France). FBS was heat-inactivated at 56 °C for 30 min. Cells were maintained at 37 °C in a humidified atmosphere containing 5% CO_2_ and were subcultured every 2 or 3 days.

The virulent streptomycin-resistant SL1344 strain of ST was kindly provided by Dr. Philippe Méresse (INSERM U1068, Marseille, France). Bacteria were grown in Luria–Bertani (LB) broth at 37 °C without shaking until mid-log phase (OD_600_ ≈ 0.6), washed in phosphate-buffered saline (PBS), and resuspended to the desired concentration. Viable counts were determined by plating serial dilutions on LB agar and then counting colony-forming units (CFUs).

*Saccharomyces boulardii* CNCM I-745 was obtained as a commercial formulation (Ultra-Levure^®^, Biocodex, Gentilly, France). The lyophilized yeast was reconstituted under sterile conditions and inoculated into Halvorson minimal medium supplemented with 2% (*w*/*v*) glucose. To ensure aerobic growth, cultures were grown overnight at 37 °C with shaking. Yeast cells were then harvested by centrifugation, washed with sterile phosphate-buffered saline (PBS), and adjusted to 10^8^ CFU/mL in DMEM before use.

### 2.2. The In Vitro Experimental Protocols

RAW264.7 cells were maintained routinely in 100 mm culture plates. After detachment, the cells were resuspended, and the cell number was determined using Malassez’s hemocytometer. Cell viability (distinguishing viable cells from dead cells and debris) was assessed in a Trypan Blue exclusion assay. The appropriate volume of cell suspension was then calculated for the seeding of 5 × 10^5^ viable cells per six-well plates and incubation overnight. Prior to exposure to ST and *S. boulardii*, the cells were washed and maintained in antibiotic-free DMEM and supplemented with 2 mM L-glutamine and 10% heat-inactivated FBS.

Bacteria grown overnight in LB broth were pelleted, resuspended in DMEM to a multiplicity of infection (MOI) of 10. Where appropriate, 1 × 10^7^ *S. boulardii* cells were added per well.

**Figure 1:**
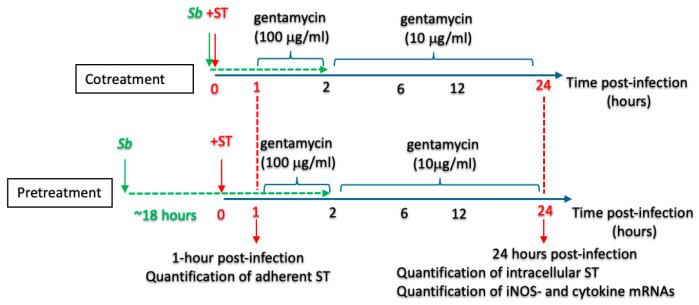
The pretreatment and cotreatment protocols. A schematic representation of the treatment timelines applied to RAW264.7 macrophage-like cells. In the pretreatment protocol, the RAW264.7 cells were exposed to the probiotic *S. boulardii* (*Sb*) for 18 h prior to ST infection and then during the infection. In the cotreatment protocol, the cells were exposed to *S. boulardii* during the ST infection only. This design enabled the assessment of both pre-conditioning and the immediate effects of the yeast on host–pathogen interactions.

Two treatment protocols were applied (Figure 1): (i) A cotreatment protocol in which RAW264.7 cells were exposed concomitantly to ST (at an MOI of 10) and *S. boulardii* (1 × 10^7^ cells/well) for one hour, and (ii) a pretreatment protocol in which the RAW264.7 cells were additionally treated overnight (~18 h) with *S. boulardii* (1 × 10^7^ yeast cells/well) prior to exposure to ST (at an MOI of 10). It should be noted that in the present study, the term “pretreatment” was considered to additionally include the cotreatment phase, i.e., the same *S. boulardii* used for cell treatment before the ST infection remained present during the ST infection. We assessed macrophage viability under our experimental conditions and confirmed that exposure to *Saccharomyces boulardii* CNCM I-745 at the doses used in this study did not induce cytotoxicity, as determined by the absence of increased lactate dehydrogenase (LDH) release (Supplementary Figure S1).

### 2.3. Adhesion, Phagocytosis, and Bacterial Killing Assays

Following 1 h incubation with ST, the RAW264.7 cells were washed thoroughly with PBS and lysed immediately using 0.1% Triton X-100 in PBS (to quantify adherent and intracellular bacteria, Sigma-Aldrich, St. Louis, MO, USA). Lysates were serially diluted and plated on LB agar. CFUs were counted after overnight incubation at 37 °C.

A gentamicin protection assay was used to estimate intracellular survival of ST. Since gentamicin did not concentrate inside the cells, intracellular bacteria survived the incubation, but adherent and extracellular bacteria were killed. After 1 h of infection of the RAW264.7 cells, extracellular ST were killed by incubation with 100 µg/mL gentamicin in PBS for 1 h. The PBS was then replaced with 10 µg/mL gentamicin in PBS, and the cells were incubated for ~22 h. At 24 h post-infection, macrophages were lysed with 0.1% Triton X-100, and CFUs from serial dilutions were counted.

### 2.4. Gene Expression Analysis (RT-qPCR)

At 24 h post-infection, total RNA was extracted from RAW264.7 cells using TRIzol™ reagent (Invitrogen, Cergy Pontoise, France). One microgram of RNA was reverse-transcribed using the RevertAid First Strand cDNA Synthesis Kit (K1622, Thermo Scientific, Courtaboeuf, France), according to the manufacturer’s instructions. Quantitative PCRs were performed using SYBR Green Master Mix (Applied Biosystems, Villebon-sur-Yvette, France) in optical 96-well plates and analyzed with the StepOne Software v2.1 (Applied Biosystems). Primer sequences are listed in Supplementary Table S1. Gene expression was analyzed using the comparative threshold cycle (Ct) method (Applied Biosystems). For each sample, the Ct value of the target gene was normalized against the Ct value of the housekeeping gene 36B4 (Rplp0), which was used as an internal control. Relative gene expression was calculated using the ΔCt method (Ct_target − Ct_36B4). For comparisons of experimental vs. control conditions, relative quantification was determined using the ΔΔCt method, and fold changes were calculated as 2^−ΔΔCt^.

In comparisons of experimental conditions the gene of interest’s expression level under the control condition was always set to 1.

### 2.5. Statistical Analysis

Results are presented as the mean ± standard error of the mean (SEM). Data were analyzed using GraphPad Prism software (version 8, GraphPad Software LLC., Boston, MA, USA), using a one-way analysis of variance with Tukey’s test for multiple comparisons. The threshold for statistical significance was set to *p* < 0.05.

## 3. Results

### 3.1. Treatment with S. boulardii Is Associated with Lower Survival of ST Inside RAW264.7 Cells

**Figure 2:**
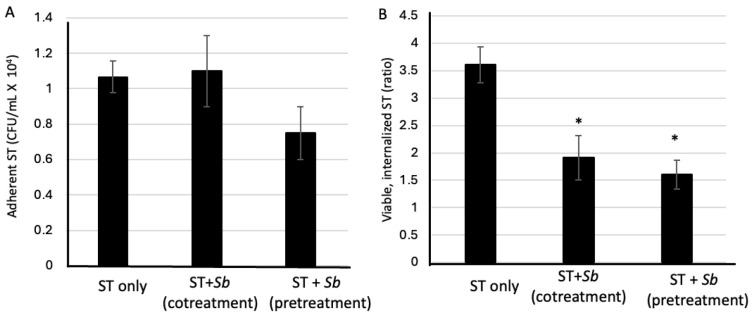
The influence of exposure to *S. boulardii* on ST adhesion and intracellular survival in RAW264.7 macrophages. (**A**). RAW264.7 cells exposed to *S. boulardii* (*Sb*, whether via cotreatment or pretreatment) and nontreated cells did not differ significantly in the CFU counts of adherent bacteria at 1 h post-infection; this indicates that the yeast did not influence the initial bacterial attachment or uptake. (**B**). 24 h post-infection, both cotreatment and pretreatment protocols were associated with a significant reduction in intracellular bacterial survival (calculated as the ratio of intracellular bacteria at 24 h post-infection to adherent bacteria at 1 h post-infection). The pretreatment protocol showed a stronger effect. The data are presented as the mean ± SEM from 3 independent experiments. * *p* < 0.05 vs. ST alone.

In a previous study, we had found that the pretreatment of mice with *S. boulardii* before infection with ST significantly decreased the invasion of the intestinal epithelium by the pathogen [29]. To determine whether *S. boulardii* modulates ST adhesion or the intracellular survival of ST, RAW264.7 cells were either treated with *S. boulardii* prior to and during an ST infection at an MOI of 10 (the pretreatment protocol), or treated with *S. boulardii* solely during an ST infection (the cotreatment protocol) (for details see Figure 1). ST adhesion to macrophages was not significantly affected by either treatment with *S. boulardii*. The CFU counts 1 h post-infection were similar to those observed for nontreated controls (*p* > 0.05; Figure 2A); thus, *S. boulardii* does not interfere with the initial bacterial binding.

In contrast, intracellular bacterial survival (as assessed using a gentamicin protection assay) 24 h post-infection was significantly lower in both *S. boulardii*-treated groups than in nontreated, ST-infected control cells (*p* < 0.05) (Figure 2B). The difference was greater for the pretreatment protocol, which indicates that prior exposure to *S. boulardii* enhances the macrophage-like cells’ bactericidal capacity. These findings suggest that *S. boulardii* enhances the intracellular killing of *Salmonella* without affecting the initial adhesion.

### 3.2. Treatment with S. boulardii Is Associated with Greater iNOS Expression in Infected RAW264.7 Cells

**Figure 3:**
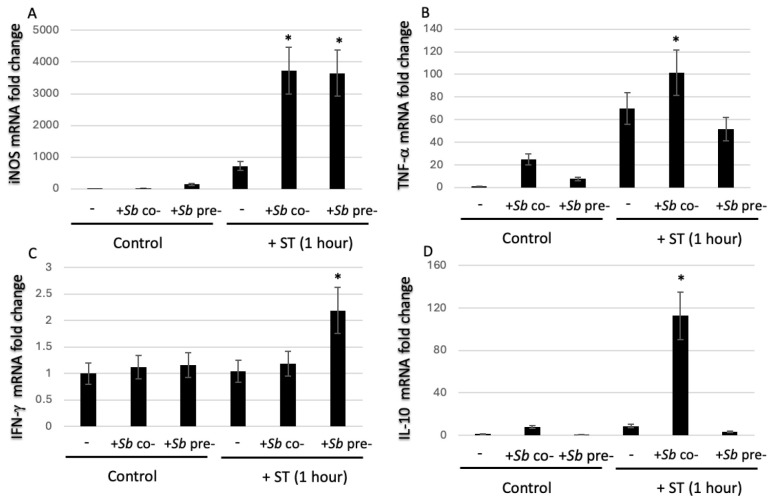
The influence of exposure to *S. boulardii* on the expression of iNOS and cytokines. Using RT-qPCR assays, mRNA expression levels of (**A**) *iNOS*, (**B**) *TNF-α*, (**C**) *IFN-γ*, and (**D**) *IL-10* were quantified 24 h post-infection in ST-infected RAW264.7 cells alone, after cotreatment with *S. boulardii* (“ST + *S.b* cotreat.”), after pretreatment with *S. boulardii* (“ST + *S.b* pretreat”) or after overnight exposure to *S. boulardii* alone in the absence of ST. Fold changes are expressed relative to nontreated controls. * *p* < 0.05 vs. ST alone. The data are presented as the mean ± SEM from 3 independent experiments.

Given the central role of iNOS in the macrophages’ antimicrobial defenses, we quantified iNOS mRNA levels in RAW264.7 cells infected with ST in the presence or absence of *S. boulardii* (Figure 3A). Treatment with *S. boulardii* alone did not alter iNOS gene expression, relative to nontreated control cells. As expected, ST infection significantly upregulated iNOS expression. Relative to ST infection alone, iNOS expression 24 h post-infection was higher after pretreatment or cotreatment with *S. boulardii*. These results indicate that exposure to *S. boulardii* enhances iNOS expression and suggests that the NO pathway contributes to the yeast’s antimicrobial influence on macrophages.

### 3.3. Treatment with S. boulardii Modulates the Expression of Pro- and Anti-Inflammatory Cytokines

Whereas cytokines such as TNF-α and IFN-γ are key inducers of iNOS gene expression, IL-10 suppresses this expression [18,20]. We evaluated the expression of these pro- and anti-inflammatory cytokines in the supernatant of RAW264.7 cell cultures following treatment with *S. boulardii* and/or ST infection (Supplementary Figure S2). 24 h post-infection, protein levels of IL-10 and TNF-α were lower in the supernatant of cells pretreated with *S. boulardii*. These findings prompted us to quantify cytokine expression in qPCR assays.

After ST infection, mRNA levels of IFN-γ and/or TNF-α were generally higher after *S. boulardii* treatment; this indicated an enhancement of the RAW264.7 cells’ pro-inflammatory response. However, the differences varied from one cytokine to another and depended on the treatment context. TNF-α expression was significantly higher in cells exposed to *S. boulardii* than in control cells (Figure 3B). As expected, TNF-α expression was significantly higher in ST-infected cells than in control, noninfected cells (whether exposed to *S. boulardii* or not). Relative to ST-infected cells, TNF-α expression was significantly higher 24 h post-infection in the *S. boulardii* cotreatment protocol but not in the pretreatment *S. boulardii* protocol (Figure 3B). IFN-γ mRNA expression was significantly greater with the pretreatment protocol than with all the other conditions, including the cotreatment protocol (Figure 3C). IFN-γ expression was not upregulated following exposure to *S. boulardii* alone. Lastly, IL-10 expression was upregulated with the cotreatment protocol, which might have reflected an inflammation-limiting feedback mechanism (Figure 3D). In contrast, the downregulation of IL-10 expression in the pretreatment protocol was consistent with a more pro-inflammatory, activated macrophage phenotype.

### 3.4. Confirmation of Invasion Data and Modulation of Cytokine Expression in the Intestine of ST-Lux–Infected Mice Treated with S. boulardii

In a previous study published by our group [29], a streptomycin-treated mouse model infected with a bioluminescent *Salmonella enterica* serovar Typhimurium strain (ST-lux) was used to monitor bacterial progression along the intestinal tract. This study demonstrated that pretreatment of mice with *Saccharomyces boulardii* accelerated bacterial transit and increased fecal elimination. In addition, *S. boulardii* pretreatment significantly reduced the bacterial invasion of the intestinal epithelium and modulated cytokine expression, as evidenced by increased IFN-γ and decreased IL-10 mRNA levels in the intestine [28,29].

**Figure 4:**
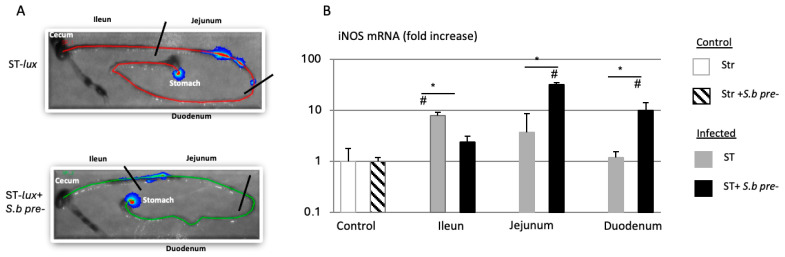
The upregulation of iNOS expression in the mouse. (**A**) *In vivo* bioluminescence imaging of intestinal tracts from mice 45 min post-infection with ST-lux alone or after cotreatment with *S. boulardii (Sb)*. (**B**) RT-qPCR assays of *iNOS* mRNA expression in the ileum, jejunum, and duodenum 45 min post-infection with ST-lux alone or after pretreatment with *S. boulardii*. The data are representative of five independent experiments, # *p* < 0.05 treated animal vs. control, * *p* < 0.05 ST alone vs. ST + *S.b* For the materials and methods, and statistical analysis see [29].

In the present study, it is further shown that *S. boulardii* treatment is associated with increased iNOS gene expression in different regions of the intestine (Figure 4). These previously unpublished *in vivo* data support the *in vitro* findings obtained in RAW264.7 macrophages and suggest that *S. boulardii* may contribute to enhanced antimicrobial responses, at least in part, through the modulation of macrophage activation.

## 4. Discussion

The results of the present study demonstrate that *S. boulardii* CNCM I-745 significantly enhances the antimicrobial capacity of RAW264.7 macrophage-like cells infected with ST. *Saccharomyces boulardii* did not interfere with the adhesion or internalization of the bacteria, as shown by similar CFU counts 1 h post-infection in the presence vs. the absence of the yeast. However, treatment with *S. boulardii* (and particularly when a pretreatment protocol was applied) was associated with significantly lower levels of intracellular bacterial viability at later time points post-infection. The fact that this effect was concomitant with greater expression of iNOS gene expression suggests that *S. boulardii* enhances macrophage antimicrobial mechanisms by activating NO synthesis. At this stage of the investigation, nitric oxide production was not directly measured and the association between reduced *Salmonella* survival and the modulation of the iNos patway should be considered correlative in nature.

In *Salmonella* infections, two of the key features are (i) the invasion of non-phagocytic epithelial cells in the lamina propria and the subsequent induction of apoptosis, and (ii) the invasion, survival, and replication of bacteria within macrophages. In previous research, we demonstrated that treatment with *S. boulardii* was not associated with a difference in the number of *Salmonella* adhering to T84 epithelial cells. However, when T84 cells were co-infected with *S. boulardii* and ST, the number of viable intracellular bacteria was significantly lower, especially after pretreatment with the yeast [28]. Our present results confirm these findings and extend them to ST-infected RAW264.7 macrophage-like cells; treatment with *S. boulardii* did not influence bacterial adhesion but was associated with significantly lower intracellular viability of the pathogen.

Our *in vitro* results are consistent with the literature data generated in two murine models. In a typhoid-like model, the administration of *S. boulardii* to infected mice abolished the translocation of viable *Salmonella* to the liver [28]. Similarly, in a streptomycin-pretreated model mimicking gastroenteritis, treatment with *S. boulardii* was associated with less bacterial dissemination to the liver and spleen [29]. One can reasonably hypothesize that *in vivo*, the killing of intracellular bacteria by macrophages might be an important mechanism by which *S. boulardii* limits the systemic dissemination of *Salmonella* sp.

The NO produced by iNOS is a central effector in the macrophage responses to microbes in general and to intracellular pathogens (such as *Salmonella* sp.) in particular [17,32]. Our results show that *S. boulardii* upregulates iNOS expression in RAW264.7 macrophages infected with ST. As iNOS is primarily regulated at the transcriptional level by cytokines and microbial products [17], we assessed the expression of IFN-γ and TNF-α; these cytokines act synergistically to induce iNOS transcription and enhance macrophage antimicrobial activity [18,20]. Microbial ingestion often triggers autocrine production of these cytokines [33,34]. We also measured the expression of IL-10, a potent downregulator of iNOS expression through suppression of TNF-α signaling [19]

Our experiments highlighted the context-dependent modulation of pro- and anti-inflammatory cytokines by *S. boulardii*, depending on whether the yeast is administered as a pretreatment or cotreatment. Pretreatment was associated with higher IFN-γ expression and lower IL-10 expression, i.e., a shift towards a more pro-inflammatory, antimicrobial macrophage phenotype. In contrast, cotreatment was associated with the upregulation of both TNF-α and IL-10, which might correspond to a more balanced inflammatory response that limits excessive inflammation once an infection has been established. This dual response underscores *S. boulardii*’s dynamic immunomodulatory capacity as a function of the timing of the infection. The *in vivo* relevance of cytokine modulation has been observed in streptomycin-treated mice, where *S. boulardii* upregulated IFN-γ expression and downregulated IL-10 expression in the intestinal mucosa (relative to ST-infected controls) [29]. This cytokine profile is consistent with an environment that is conducive to iNOS-mediated bacterial clearance.

Although the cytokine profile and iNOS upregulation observed here are consistent with enhanced antimicrobial activity, a direct NO-dependent mechanism cannot be concluded without pharmacological inhibition studies. Confirmation of these findings and their functional relevance *in vivo* will therefore be required.

In addition to NO–mediated antimicrobial activity, macrophages use several complementary mechanisms to control intracellular pathogens like ST. These include the generation of reactive oxygen species that contribute to bacterial killing and act in coordination with reactive nitrogen species to enhance antimicrobial efficacy. Previous studies have shown that probiotic microorganisms (including *Saccharomyces boulardii* CNCM I-745) can modulate the host’s innate immune responses and intracellular signaling pathways. Therefore, in addition to the transcriptional upregulation of iNOS observed in the present study, it is possible that *S. boulardii* also influences other antimicrobial pathways (such as reactive oxygen species generation and autophagy) and thereby contributes to enhanced bacterial clearance. Further studies will be required to determine the relative contribution of these mechanisms and to fully elucidate the pathways involved in the *S. boulardii*’s protective effects on macrophages.

The present study had a number of strengths. Firstly, the RAW264.7 cell line is a very well characterized macrophage model. Secondly, our *in vitro* results confirmed the *in vivo* observations made previously in a mouse model. Thirdly, the CNCM I-745 strain of *S. boulardii* is present in probiotic formulations used widely with preventive and curative intent in human health.

The study also had limitations, most of which were inherent to its *in vitro* design. It remains to be seen whether our findings can be extended to native human macrophages and, indeed, other components of the human immune system that are known to be influenced directly or indirectly by *S. boulardii*. Further studies are required to better define the mechanistic basis of the observed effects, including comparisons between live yeast and yeast-conditioned medium. Such approaches would help to distinguish between the direct contact-dependent effects of yeast cells and those mediated by soluble factors released into the medium.

## 5. Conclusions

Our present *in vitro* findings suggest that *S. boulardii* contributes to host defenses by enhancing macrophage activation, modulating cytokine expression, and promoting NO–related antimicrobial mechanisms. The more pronounced effects observed with our pretreatment protocol suggest that prior exposure to *S. boulardii* may prime innate immune transcriptional responses and potentially improve the cellular response to subsequent bacterial challenge.

## Data Availability

The data presented in this study are available on request from the corresponding author due to privacy reason.

## Supplementary Materials

The following supporting information can be downloaded at: https://www.mdpi.com/article/10.3390/microorganisms14040787/s1, Figure S1: Pretreatment of RAW264.7 cells with *S. boulardii* idid not induced cytotoxicity; Figure S2: Levels of pro- and anti-inflammatory cytokines in the supernatant of RAW264.7 cells; Table S1: Murine primer sequences used in the RT-qPCR assays.

## Abbreviations

The following abbreviations are used in this manuscript:

ST	*Salmonella enterica* serovar Typhimurium
iNOS	inducible nitric oxide synthase
TNF-α	tumor necrosis factor alpha
IL	interleukin
IFN-γ	interferon gamma
NO	nitric oxide
SPI	*Salmonella* pathogenicity island
FBS	fetal bovine serum
LB	Luria–Bertani
CFU	colony-forming unit
DMEM	Dulbecco’s Modified Eagle Medium
MOI	multiplicity of infection
PBS	phosphate-buffered saline
SEM	standard error of the mean
