# Supplementary material for: Saccharomyces boulardii CNCM I-745 Stimulates Intracellular Antimicrobial Activity Against Salmonella Typhimurium in Murine Macrophages
Source: Microorganisms. 2026 Mar 31;14(4):787. doi: 10.3390/microorganisms14040787 (PMC13118339; doi:10.3390/microorganisms14040787)
Supplement: Supplementary file 1 [file microorganisms-14-00787-s001.zip › microorganisms-4138160-supplementary.pdf]

Supplementary Table S1. Murine primer sequences used in the RT-qPCR assays.

Gene	Forward primer	Reverse primer
36B4	TCCAGGCTTTGGGCATCA	CTTTATCAGCTGCACATCACTCAGA
iNOS	CATTGGAAGTGAAGCGTTTCG	CAGCTGGGCTGTACAAACCTT
TNF-α	AGGCTGCCCCGACTACGT	GACTTTCTCCTGGTATGAGATAGCAAA
IFN-γ	CAGCAACAGCAAGGCGAAA	CTGGACCTGTGGGTTGTTGAC
IL-10	CTGGACCTGTGGGTTGTTGAC	ACCTGCTCCACTGCCTTGCT

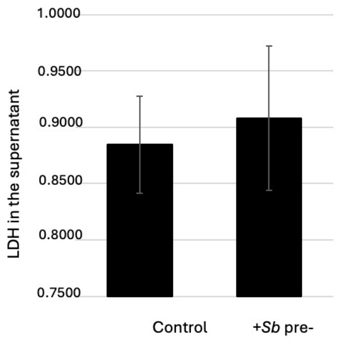

Supplementary Figure S1. Pretreatment of RAW264.7 cells with *S. boulardii* idid not induced cytotoxicity. LDH released into the cell culture supernatant (measured with Cytotoxicity Detection Kit (Takara Bio, Saint-Germain-en-Laye, France).was performed in control RAW264.7 cells and cells pretreated ~18 hours with *S. boulardii* (“*S.b* pre-”). n=3 independent experiments.

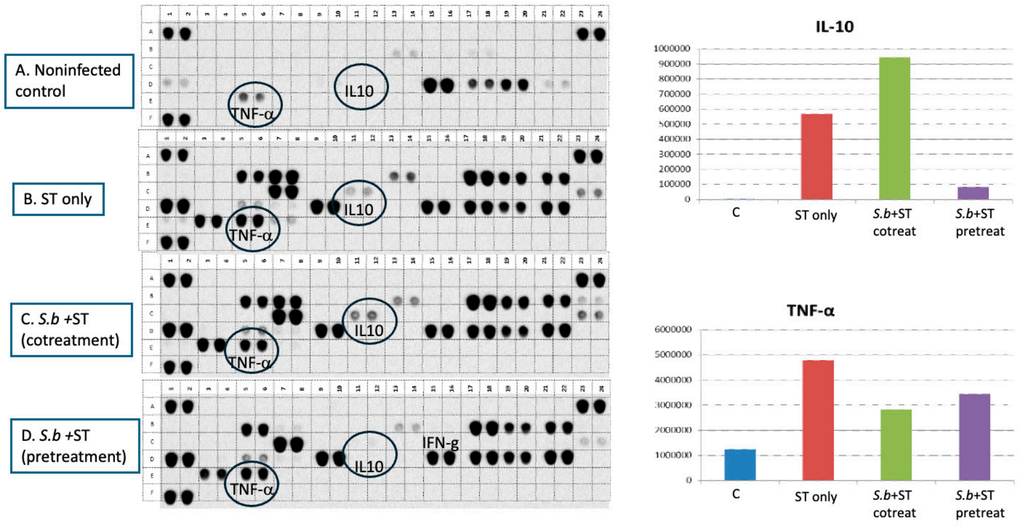

Supplementary Figure S2. Levels of pro- and anti-inflammatory cytokines in the supernatant of RAW264.7 cells. In (A) control RAW264.7 cells and (B) RAW264.7 cells infected with ST alone, (C) cotreated with S. boulardii (i.e. during the ST infection only: “ST+Sb cotreat”) or (D) pretreated with S. boulardii (i.e. before and during the ST infection: “ST+Sb pretreat”) (D). Cytokine levels were assessed by using the Proteome Profiler Mouse cytokine Array (Kit A from R&S systems cat. Number ARY006) and were quantified by measuring the intensity of the corresponding spot.
